# Progress of Epidemics in Thirty-Nine Towns and Districts

**Published:** 1857-04

**Authors:** 


### PROGRESS OF EPIDEMICS.

### LOCAL REPORTS OF EPIDEMIC AND ENDEMIC DISEASES

*During the Months of December, January, and February,* 1856-57.

| Place. | County. | Lat. | Long. | Observer. |
|---|---|---|---|---|
| St. Mary, Scilly | Cornwall - | 49.50 N. | 6.18 W. | J. G. Moyle, Esq. |
| Teignmouth - | Devonshire - | 50.32 N. | 3.29 W. | W. C. Lake, Esq. |
| Canterbury - | Kent - - | 51.17 N. | 1. 4 E. | G. Rigden, Esq. |
| Chatham - | Kent - - | 51.21 N. | 0.14 E. | F. J. Brown, M.D. |
| Wandsworth - | Surrey - - | 51.28 N. | 0. 7 W. | G. E. Nicholas, Esq. |
| Putney - - | Surrey - - | 51.28 N. | 0. 8 W. | R. H. Whiteman, Esq. |
| Up. Holloway | Middlesex - | 51.32 N. | 0.03 E. | W. B. Kesteven, Esq. |
| Wanstead - | Essex - - | 51.32 N. | 0. 2 W. | F. Collins, M.D. |
| Swansea - | Glamorgansh. | 51.38 N. | 3.50 W. | W. H. Michael, Esq. |
| Aspley Guise - | Bedfordshire - | 52. 1 N. | 0.37 W. | J. Williams, M.D. |
| Saffron Walden | Essex - - | 52. 3 N. | 0.12 E. | T. Spurgin, Esq.<br>H. Stear, Esq. |
| Bedford - - | Bedfordshire - | 52. 8 N. | 0.51 W. | T. H. Barker, M.D. |
| Sharnbrook - | Bedfordshire - | 52.12 N. | 0.40 W. | R. S. Stedman, Esq. |
| Newpt. Pagnell | Buckinghams. | 52.10 N. | 0.42 W. | G. O. Rogers, Esq. |
| Wellingbro' - | Northamptons. | 52.20 N. | 0.40 W. | B. Dulley, Esq. |
| Stourbridge - | Worcestersh. - | 52.28 N. | 2.32 W. | A. Freer, Esq. |
| Beccles - - | Suffolk - - | 52.25 N. | 1.48 E. | W. E. Crowfoot, Esq. |
| Thetford - | Norfolk - - | 52.26 N. | 0.45 E. | H. W. Bailey, Esq. |
| Dudley - - | Staffordshire - | 52.30 N. | 2.10 W. | J. H. Houghton, Esq. |
| Barrowden - | Rutland - | 52.34 N. | 0.38 W. | H. J. Swann, Esq. |
| Wisbeach - | Cambridgesh. | 52.39 N. | 0. 5 E. | W. H. Hole, Esq. |
| East Dereham | Norfolk - - | 52.40 N. | 0.57 E. | J. Vincent, M.D. |
| Pontesbury - | Shropshire - | 52.43 N. | 2.50 W. | Wm. Eddowes, Esq. |
| Nottingham - | Nottinghamsh. | 52.50 N. | 1.10 W. | T. Robertson, M.D. |
| Burton-on-Trt. | Staffordshire - | 52.53 N. | 1.53 W. | S. Thomson, M.D. |
| Oswestry - | Shropshire - | 52.53 N. | 2.59 W. | P. Cartwright, Esq. |
| Wrexham - | Denbighshire - | 53. 2 N. | 3. 1 W. | E. Williams, M.D. |
| Hawarden - | Flintshire - | 53.11 N. | 3. 2 W. | T. Moffat, M.D. |
| Lincoln - | Lincolnshire - | 53.12 N. | 0. 5 W. | S. Lowe, Esq. |
| Alford - - | Lincolnshire - | 53.15 N. | 0. 6 E. | R. U. West, M.D. |
| Staveley - | Derbyshire - | 53.15 N. | 1.20 W. | G. B. Thorpe, Esq. |
| Gainsborough | Lincolnshire - | 53.23 N. | 0.47 W. | D. Mackinder, M.D. |
| Liverpool - | Lancashire - | 53.24 N. | 2.59 W. | Thos. Bickerton, Esq. |
| Warrington - | Lancashire - | 53.25 N. | 2.32 W. | C. N. Spinks, Esq. |
| Bolton - - | Lancashire - | 53.35 N. | 2.19 W. | W. H. Pendlebury, Esq. |
| York - - | Yorkshire - | 53.58 N. | 1. 3 W. | W. Procter, Esq. |
| Wst. Auckland | Durham - | 54.45 N. | 1.40 W. | G. Todd, Esq. |
| Rothbury - | Northumberld. | 55.25 N. | 1.50 W. | E. C. Summers, Esq. |
| Lerwick - | Shetland Isles | 60. 9 N. | 1. 8 W. | G. W. Spence, M.D. |

### QUARTERLY STATEMENT—No. IX.

[*The dates denote that the disease appeared in the weeks then ending.*]

SCARLET FEVER.

Teignmouth..December 5
Canterbury..All Dec. and Jan.
Chatham..December 12
Wandsworth..All Dec., Jan. 2, 30, Feb. [20-27
Putney..December 5-12
Upper Holloway..Jan. 9-30, Feb. 6
Swansea..December 5, 26
Aspley Guise..Dec. 5, 19, Jan. 9
Saffron Walden..January 30
Sharnbrook..December 5
Newport Pagnell..Dec. 19, Jan. 16-30, [Feb. 6

Stourbridge..Jan. 30, Feb. 6
Beccles..December 5-12
Dudley..Jan. 2, 9, 23, 30, Feb. 13-27
Barrowden..Dec. 19, Feb. 13-20
East Dereham..All Dec., Jan. 2-23
Pontesbury..Every week
Alford..January 9-16
Gainsborough..All Jan. and Feb.
Liverpool..Every week [6, 13, 27
Warrington..Dec.5-26, Jan. 2, 30, Feb.
Bolton-le-Moors..Dec. 5-19, Jan. 2, 9,
West Auckland..All Jan. & Feb. [23

MEASLES.

Teignmouth..December 5-12
Canterbury. January 16-23
Chatham..December 5
Wandsworth..Jan. 2, 9, 23, 30, Feb. 13
Putney..February 20
Upper Holloway..All Dec., Jan. 2, all
Wanstead..Jan. 9-30, all Feb. [Feb.
Saffron Walden..Jan. 9, all Feb.
Bedford..Every week
Thetford..December 19
Wisbeach..All December & January
Pontesbury..Every week
Burton-on-Trent..December 5-12
Wrexham..December 19-26
Lincoln..All Jan., Feb. 6-13
Alford..Dec. 26, all Jan. and Feb.
Gainsborough..February 13-27
Liverpool..Every week
Warrington..Jan. 30, Feb. 6-20 [Feb.
Bolton-le Moors..Dec.12-19, all Jan. &
York..Dec. 12-19, Jan. 2, 16, all Feb.
West Auckland..All December

SMALL POX.

Chatham..December 5, 26
Stourbridge..February 13-20
Dudley..All Dec. and Jan., Feb. 6, 20
Wisbeach..February 20-27
East Dereham..February 27
Pontesbury..January 2-23
Oswestry..Dec. 26, all Jan., Feb. 6-20
Wrexham..December 5
Gainsborough..All February
Liverpool..Every week
Warrington..Feb. 13-20 [20-27
Lerwick..Dec. 5-19, Jan. 9, 16, 30, Feb.

HOOPING COUGH.

Teignmouth..December 5
Canterbury..Every wk. except Feb. 27
Chatham..Jan. 30, Feb. 20-27
Wandsworth..Every wk. except Feb. 27
Upper Holloway..All February
Wanstead..January 9-16
Swansea..Every week
Saffron Walden..February 13
Bedford..Every week
Sharnbrook..All February
Newport Pagnell..Jan. 30, all Feb.
Stourbridge..February 6, 20
Beccles..Jan. 9-30, all Feb.
Thetford..Dec. 26, Jan. 16, Feb. 13
Barrowden..All Dec., Jan. 2-9, all Feb.
Wisbeach..All February
Pontesbury..Every week
Burton-on-Trent..January 9
Oswestry..Every week except Feb. 27
Liverpool..Every week
Warrington..Every week
Bolton-le-Moors..Dec. 5, 12, 26, Jan. 2, 16-30, February 6 13
York..Dec. 5-19, Jan. 9, 23, Feb. 13
West Auckland..All December

CROUP.

St. Mary's, Scilly..Jan. 23-30, all Feb.
Teignmouth..January 23
Chatham..January 2
Wanstead..December 19-26
Swansea..Dec. 12, Jan. 9-30
Bedford..December 19, January 16
Stourbridge..Jan. 23, Feb. 6, 20
Thetford..February 6-13
Dudley..February 6
Burton-on-Trent..February 27
Wrexham..Dec. 5, Jan. 2-9, Feb. 6
Alford..February 13
Liverpool..All Dec., Jan. 2-16, Feb. 20
Warrington..February 13
Bolton-le-Moors..February 13, 27
York..Dec. 12-19, Feb. 6-13

CATARRH.

St. Mary's, Scilly..February 13-27
Teignmouth..Dec. 5, 19, 26, Jan. 2-23, February 13-20
Chatham..All Dec. & Jan., Feb. 6-13
Wandsworth..Dec. 5, 26, Jan. 9-23,
Putney..Every week [Feb. 6-20
Upper Holloway..Jan. 30, Feb. 6-13
Swansea..Every week [20 27
Aspley Guise..Dec. 5, 26, Jan. 2, Feb.
Saffron Walden..Dec. 5-26, Jan. 9, all
Bedford..Jan. 2, 9, 30 [Feb.
Sharnbrook..All Dec. & Jan., Feb. 6
Newport Pagnell..All Dec., Jan. 9-23,
Stourbridge..Feb. 6 [Feb. 13-27
Beccles..Dec.19-26, Jan. 2-16, Feb. 6-20
Thetford..All Dec., Jan. 16-30, Feb.
Barrowden..December 5 [13-27
Wisbeach..Every week
Burton-on-Trent..Every week
Oswestry..Dec. 12-26, Jan. 2-23
Alford..Jan. 2, 23, 30, Feb. 6
Gainsborough..All December
Liverpool..Every week
Warrington..All Dec., January 23-30, February 6-20
Bolton-le-Moors..Dec. 12-19, Jan. 2, 9, 23, 30, all February
Lerwick..All Dec., Jan. 2-9, Feb. 6-13

INFLUENZA.

Teignmouth..Dec.5-19,all Jan.,Feb.6,
Chatham..All Dec.&Jan.,Feb.6 [13,27
Wandsworth..Jan. 16, Feb. 13-20
Wanstead..All Dec. & Jan., Feb. 6
Swansea..Dec. 12-19, Jan. 16
Aspley Guise..January 16, 30
Saffron Walden..Every week
Bedford..Jan. 23, Feb. 6-20
Newport Pagnell..Every week
Stourbridge..All February
Beccles..December 5
Thetford..All Dec., Jan. 2, 9, 23, 30,
Dudley..Feb. 20 [Feb. 27
Barrowden..December 19
Wrexham..Dec.5,Jan.9-23,Feb.13-27
Lincoln..All February
Alford..February 13-27
Liverpool..All December
Bolton le-Moors..Jan.9,23,30,Feb.6
York..Dec.5,12,26,Jan.16-30,Feb.20
Lerwick..All Dec., Jan, 2, 9, 23, 30,
February 6, 20

ERYSIPELAS.

Teignmouth..December 5
Canterbury..December 5
Chatham..All Dec.,Jan.9,16,Feb.6,27
Wandsworth..Jan.9,16,30,Feb.13,27
Putney..February 6
Wanstead..December 26
Swansea..December 5 [Feb.6,20
SaffronWalden..Dec.5,26,Jan.16,23,
Sharnbrook..Dec. 5, Jan. 9, 23, Feb. 6
Newport Pagnell..Jan. 2, 16-30, Feb.
Beccles..Jan. 30, Feb. 6 [6-20
Thetford..Dec. 5, 19, 26, Jan. 23, Feb.
Dudley..Jan. 9, Feb. 13 [20-27
Wisbeach..January 23-30
East Dereham..January 23-30
Pontesbury..Jan. 2-16, 30, Feb. 6-13
Burton-on-Trent..February 13
Hawarden..Dec. 19, Jan. 2
Gainsborough..February 6-20
Liverpool..Dec. 19, Jan. 2, 9, 23, 30,
February 13-27 [13-27
Warrington..Dec. 26, Jan. 16-23, Feb.
Bolton-le-Moors..Dec.19-26,Jan.9-30,
York..February 6-13 [Feb. 6
Rothbury..February 27
Lerwick. Dec.5-12,Jan.16,30,Feb.27

CHOLERA.

Wrexham..February 20-27
Warrington..February 6, 20

AGUE.

Canterbury..Jan. 16-30, Feb. 12-27
Chatham..All Dec., Jan. 2-16, 30, Feb.
Swansea..December 19-26 [13-27
Bedford..Dec. 26, all Jan., Feb. 6
Sharnbrook..February 27
Newport Pagnell..January 23, 30
Thetford..Dec. 19, Jan. 23, Feb. 13-20
Barrowden..February 13
Wisbeach..Every week
Lincoln..December 5-12
Gainsborough..All Jan. and Feb.
Liverpool..Dec. 26, Jan. 2
Warrington..February 13

REMITTENT FEVER.

Teignmouth..Dec.12, Jan. 23, Feb. 20
Canterbury..Jan. 30, Feb. 6
Putney..Dec. 12, Jan. 9, 23, Feb. 6-13
Aspley Guise..December 19
Saffron Walden..Dec. 12-26 [Feb. 6
Newport Pagnell..All Dec., Jan. 2-16,
Beccles..Dec. 5-12, Jan. 9-30, Feb. 6,
Wisbeach..Feb. 13-27 [20, 27
Oswestry..All February
Wrexham..February 13-20
Alford..February 27
Gainsborough..February 13-27
Liverpool..Dec.5-19, Jan.9,23, Feb.6,
Warrington..Jan. 26 [20, 27
Bolton-le-Moors..Dec. 12-26, Jan. 2,
16-30, February 6-20
York..Jan. 16, Feb. 20
Rothbury..Jan. 30, all February

DIARRHŒA.

Teignmouth..Dec. 12-26, Jan. 2, 30
Chatham..Dec. 5-19, Jan. 2, 9, 30, Feb.
Wandsworth..Jan. 16, all Feb. [6-20
Putney..Dec. 12-26, Jan. 2, 23, 30
Swansea..Dec.5-19, Jan.16, Feb.13-27
Aspley Guise..Dec. 5-12, Jan. 16-30,
February 6 [Feb. 6, 27
Saffron Walden..Dec. 5-19, Jan. 30,
Bedford..Dec.19-26, Jan.2-16,30, Feb.
Sharnbrook..Every week [13, 27
Newport Pagnell..Dec. 5, 19, 26, all
Stourbridge..Feb. 20 [Jan.&Feb.
Beccles..Dec. 5, 12, 26, Jan. 16-23
Thetford..All Dec., Jan.23-30, Feb.6,27
Dudley..Dec.5-19, all Jan., Feb.6,20,27
Barrowden..Dec.19, Jan. 2,23-30, Feb.
East Dereham..Dec.26, Jan.2-23 [6,13
Burton-on-Trent..Dec. 5, Jan. 30,
February 6, 20 [6, 20, 27
Hawarden..Dec. 5, 19, all Jan., Feb.
Alford..Jan. 23-30, Feb. 6, 20
Gainsborough..Every week
Liverpool..Every week
York..Dec. 12-19, Jan. 30
Rothbury..Dec. 19, Jan. 16-23
Lerwick..January 9, 23

DYSENTERY.

Teignmouth..Jan. 23, Feb. 13, 27
Chatham..Dec. 5, 26, Feb. 20-27
Wandsworth..February 13
Putney..December 5
Aspley Guise..Dec. 5, Feb. 20
Saffron Walden..January 30

Bedford..December 12-19
Newport Pagnell..Dec. 26, Jan. 2, 23, 30, February 6, 13, 27
Stourbridge..February 27
Beccles..January 9-16
East Dereham..Jan. 9 30, all Feb.
Pontesbury..January 16-30
Alford..Jan. 16, Feb. 20
Gainsborough..February 20 27
Liverpool..Every week

TYPHUS.

St. Mary's, Scilly..Every week
Canterbury..All Dec. & Jan., Feb. 6
Chatham..Jan. 23-30, all Feb.
Wandsworth..Dec. 5, 19, 26, Jan. 2-16
Putney..Jan. 9, Feb. 27
Aspley Guise..Jan. 30, Feb. 6-20
Saffron Walden..Dec. 5, Jan. 2
Bedford..Dec. 12-26, all Jan.
Sharnbrook..Jan. 16-30, Feb. 6
Beccles..Jan. 16, 30, Feb. 13
Thetford..All Dec. & Jan., Feb. 27
Dudley..All Dec. & Jan., Feb. 6, 13, 27
Wisbeach..Dec. 26, all Jan. & Feb.
East Dereham..February 20 27
Pontesbury..Every week
Lincoln..Dec. 12-26, Jan. 2
Gainsborough..All Dec. and Jan.
Liverpool..All Dec. & Jan., Feb. 6, 27
Warrington..Dec. 5-26, Feb. 6
Bolton-le-Moors..Dec. 19, Jan. 23-30, all February
Rothbury..Every week
Lerwick..February 20

PUERPERAL FEVER.

Chatham..Dec. 5, 12, 26, Jan. 2, 23, 30, Feb. 6, 27
Swansea..Dec. 12
Newport Pagnell..Jan. 9, Feb. 13
Pontesbury..February 13-27
Liverpool..All Dec., Jan. 2-16
Lerwick..January 16

CARBUNCLE.

Canterbury..Dec. 12-19, Jan. 9-23
Chatham..Dec. 19, Jan. 9-16, Feb. 6
Wandsworth..February 20-27
Saffron Walden..Dec. 5-12, Jan. 9, 23, Feb. 6
Bedford..January 2-9
Sharnbrook..Dec. 19, Jan. 30, Feb. 13-27
Beccles..January 2-16
Thetford..December 19
Dudley..Dec. 5, Jan. 2, 23
Barrowden..Jan. 9, Feb. 6
Wisbeach..February 13-27
Pontesbury..Jan. 2-16, Feb. 6-20
Burton-on-Trent..Dec. 12, Jan. 9
Gainsborough..Dec. 5-26, all Jan.
Liverpool..Dec. 26, all Jan. & Feb.
Warrington..January 16
Bolton-le-Moors..February 6
York..Dec. 12, Jan. 2, 23, Feb. 13-20
Rothbury..February 27

MUMPS.

Sharnbrook..Jan. 9-30, Feb. 6

TYPHOID FEVER.

Stourbridge..February 13, 27

ACUTE RHEUMATISM.

Wrexham..January 9-23

QUINSY.

Liverpool..All Dec., Jan. 2-16

### ADDITIONAL OBSERVATIONS.

*St. Mary's, Scilly.*—Mr. Moyle writes: "We have had no cases of importance this quarter. Typhus still lingers in one locality, and has been fatal in one instance. The mortality has been one out of nine cases. The weather has been fine during the whole month. The winds have been principally west, south, and south-east. The amount of rain gauged in February at St. Mary's was 1.72 inch. The maximum temperature for the month was 55°; the minimum, 31.5. The cases of catarrh and influenza have been unimportant. Vegetation is appearing healthily in sheltered nooks."

*Canterbury.*—Mr. Rigden writes: "There has certainly been much less sickness and consequent mortality during the last three months, than in the corresponding period of either of the two preceding years. With an evidently increased population, the deaths in the three periods have been respectively—132, 130, and 111. Hooping-cough continues to prevail. Scarlet fever seems gradually declining, and but a few cases of measles have been met with. The mortality tables shew deaths from scarlatina and purpura in December, from hooping-cough, scarlatina, fever, croup, influenza, and diarrhœa in January; and from croup, scarlet fever, fever, and purpura in February."

Mr. Haffenden furnishes the following returns, drawn up by eight observers for the epidemiological committee of the East Kent and Canterbury Medical Society:—

"*Abstract of Meteorological Observations for the Winter Quarter*, 1856-57.

| | *Dec.* | *Jan.* | *Feb.* |
|---|---|---|---|
| | Deg. | Deg. | Deg. |
| Highest temperature in the day time.... | 46 | 49 | 44 |
| Lowest.............................. | 22 | 23 | 26 |
| Mean ............................... | 44·09 | 36·19 | 36·3 |
| Highest temperature in the night ...... | 42 | 45 | 40 |
| Lowest.............................. | 20 | 23 | 21 |
| Mean ............................... | 34·473 | 33·14 | 32·3 |
| Highest reading of the barometer ...... | 30·42 | 30·17 | 30·34 |
| Lowest.............................. | 28·75 | 26·08 | 29·45 |
| Mean ............................... | 29·723 | 29·138 | 29·948 |
| Amount of rain in inches .............. | 2·22 | 2·83 | ·64 |

Direction of wind (the number indicates the number of days certain winds prevailed). *December* (only the last nineteen days observed)—N. 2, SW. 6, W. 6, NW. 5. *January*—N. 3, E. 1, SE. 1, SSE. 1, S. 3, SSW. 1, SW. 9, WSW. 1, W. 2, WNW. 1, NW. 4. *February*—NE. 2, E. 2, ESE. 1, SE. 1, SSE. 3, S. 9, SSW. 2, SW. 4, W. 4.

*Abstract of the returns of Zymotic diseases during the Winter Quarter*, 1856-57.

"Scarlatina. Fifty-two cases are reported this quarter; of these, thirty-seven occurred in the month of December, twelve in January, and three in February. The disease is reported to have been mild. There have been four deaths from it—all females; one, six years of age, had severe inflammation of the throat, and sank rapidly. Two of the age of six years, and one of two years, had the malignant form. Seven are reported to have been anasarcous, and two with albuminous urine. Hooping-cough shows a decrease this quarter. Eight cases are returned, seven of which occurred in one family. The father (a shoemaker) had been to measure a family of children, some of whom had hooping-cough; he then visited a child of his own, at the Kent and Canterbury Hospital, who shortly afterwards had the disease, and before the children at home. The first child who showed symptoms at home had been nursed on the father's knees shortly after his return to the house. Two of the eight cases died; one, a male aged three years, had pneumonia; and the other, a female twin aged one year, sank from the exhausting effects of the cough. Varicella: two cases occurred in the month of January; there has been no return of it since. Measles, which had quite disappeared in the previous quarter, returned in three patients in the month of January. Only one case of croup occurred during the quarter; it proved fatal. Fever, next to scarlatina, shews the largest number returned, viz., thirty-one. The disease appears to have been very mild in form, and no death has resulted from it. Of ague, five cases occurred, being the same number as in last quarter. The type of the disease is usually tertian. Erysipelas and erythema: six cases of the former and two of the latter were returned—all of them females. In five, the head and face were the parts affected; and in three, the feet and legs. Cynanche was very prevalent in the month of December in the neighbourhood of Canterbury. Seven cases of diphtheritic inflammation of the fauces and tonsils came under the notice of Mr. Reid. They were attended with considerable fever, depression, and swelling of the tonsils, with more or less dyspnæa, and ringing cough. The fauces, part of the mouth, and especially the tonsils, were covered with soft pasty lymph, and in some places there were appearances of ulceration. The patients recovered in about four days, with some symptoms of debility remaining. One child died, after seven days illness, with all the symptoms of gangrenous sore throat. The total number of cases of zymotic diseases that have been returned during the quarter are 118, being the smallest number in any quarter since the formation of the society. The total number of deaths were seven, viz., four from scarlatina, two from hooping-cough, and one from croup."

*Chatham.*—Dr. Brown writes: "The epidemic of metria, which was noticed in last report, has continued. It has affected almost every surgeon's practice, and has very clearly declared itself non-infectious. The cases have *all* occurred in those parts of the town exposed to the river, excepting one case of putrid fever, occurring in a woman delivered of a child in a state of decomposition after three days labour, under the care of a woman. This was not a case of epidemic metria, but one of pyæmia. The deaths have been numerous: three have died out of seven cases that I have been advised of. I examined one case that died in four days. The woman, aged 33, was confined of a seven-months' child, and was seized with a violent diarrhœa in two hours. This passed into fever. Everything was found normal in the abdomen, excepting a pint of sero-purulent fluid in the peritoneal cavity. The lungs were studded with tubercle and carbonaceous matter, and presented many small vomicæ. I know of two deaths from puerperal convulsions during the last three months, one death from hydatids and hæmorrhage in a woman twelve weeks pregnant, and one sudden death fifteen days after childbirth. Nothing was found to explain the death, excepting slight serous effusion in the pericardium. This case was not seen by me, nor was I present at the *post mortem* examination. One case of 'contagious furunculoid,' affecting the face, proved fatal."

*Wandsworth.*—Mr. Nicholas records that hooping-cough was the most prevalent, and fever the most fatal, of the zymotic diseases in this sub-district during the past quarter. Zymotics caused eleven deaths: of these deaths, three occurred from fever in the County Lunatic Asylum, which is situated nearly two miles from the town. The remainder (measles 1, hooping-cough 2, scarlet fever 3, and typhus 2) occurred *entirely* amongst the labouring class, and in houses which were either ill ventilated, over-crowded, or having defective drainage and water supply; three of them had no water supply. Diseases of the respiratory organs have been unusually prevalent and severe, especially amongst the aged.

*Putney.*—Mr. Whiteman writes: "Throughout the entire quarter catarrh has prevailed extensively. Two cases of scarlatina came under my notice in the earlier part of December, since which time, with the exception of diarrhœa of a mild character during December and a part of January, and two or three isolated cases of fever of a low type, occurring for the most part in undrained localities, there has been less disease of the zymotic class than I have witnessed for some years past. The drainage operations in the poorer localities of the town are progressing, and will, I am convinced, when completed, considerably lessen the amount of disease, especially amongst the labouring classes. A somewhat severe form of pleuropneumonia is reported to prevail amongst cattle."

*Swansea.*—Mr. Michael says, "a greater mortality than the average has prevailed during the past quarter. Hooping-cough, combined with pneumonia, has proved fatal to a large number of children, and many old persons have died. The very great and rapid changes of weather have proved particularly trying; and many persons have suffered from diseases of the respiratory passages."

*Aspley Guise.*—Dr. Williams writes: "The quarter ending February 27th has been remarkable for the suddenness of atmospheric changes, which has, no doubt, tended to produce the more than usual prevalence of epidemic disease in this locality—influenza, scarlatina, and diarrhœa. The scarlatina has been generally of a mild character, and confined to children. Two cases of latent scarlatina have occurred in one house. At the commencement of the first case I was somewhat puzzled to account for the sudden and alarming appearance of general anasarca, more particularly as no cases of scarlatina had occurred in the house or the immediate neighbourhood. At that time I had not read Dr. Copland's excellent description of this rare form of the disease; but, from the similarity of the symptoms to the dropsy following the milder forms of scarlatina, I at once thought it must be connected with scarlatina poison in the blood. If any further doubt had remained upon my mind, it was in two or three days entirely removed by the occurrence of a similar case in a younger brother of the first patient. In the first case the urine was entirely suppressed for at least forty-eight hours, and when re-established, it was bloody and nearly black for some days. There was some sore throat, which Dr. Copland states to be generally *absent* in these cases. In the course of two or three days the breath became extremely offensive, as described by Johnstone, and quoted also by Copland. Both my cases recovered, though in the first case the difficulty of breathing almost amounted to suffocation. In both cases I gave ipecacuanha and calomel freely, and a saline mixture with tartar emetic and digitalis; mustard poultices and blisters were also useful. The worst case was attended by diarrhœa, which was treated by calomel and opium. I find that fever cases do best if the diarrhœa is allowed to proceed moderately. The case of typhus occurred in a house where two fatal cases of cholera occurred in 1854, and which is unfit for human habitation."

*Saffron Walden.*—Mr. Spurgeon states that the quarter has been healthy. Winds north and north-west, with little sunshine. Vegetation feeble, and spring flowers late. Variations in temperature considerable. Mr. Stear also writes: "Influenza has been the prevailing epidemic. It was most severe (especially amongst children) during the weeks ending Februay 6 and 13. It has also extensively affected cattle and horses, some cases proving fatal from the supervention of pneumonia.

*Bedford.*—Measles and hooping-cough have prevailed throughout the quarter. Ague and the allied forms of intermittent disease have been more frequent than for some years past, owing probably to the changeable and frequent foggy weather.

*Sharnbrook.*—Mr. Stedman says: "The cases of mumps (about sixteen in number) which came under my notice were very slight, and are the first cases which have occurred since the year 1849 in this district. From inquiry among other medical men, I find that the disease occurred in several adjacent localities during the months of January and February.

In all cases it appeared (as far as I can discover) spontaneously. Ozone has been very abundant all the quarter. During the last month there has been little rain. Vegetation has commenced early."

*Stourbridge.*—Mr. Freer writes: "This report dates from Jan. 16, soon after which date the report sheet was received. The quarter now past has presented fewer cases of epidemic disease than usual. Influenza has been prevalent during the last month. Many cases of small pox have occurred in the neighbourhood, but not in the town. Two only, however, under my own care; both in vaccinated persons. One was mild and discrete; the other was confluent, and is now under treatment. The town of Stourbridge is and has been for many years singularly free from epidemics. A case of fever is very seldom to be found in it, although the immediate neighbourhood has presented many severe cases of typhoid during the greater part of the past two years. During this quarter the only epidemic affecting the lower animals has been a sort of influenza in horses."

*Thetford.*—Mr. Bailey makes the following observations: "The extraordinary fluctuations of atmospheric pressure, during the month of December, were unprecedented, the maximum being, on the 16th, 30.50; and the lowest, on the 25th, 28.85. The weather was very dull and overcast, with twelve days of rains, fogs, and mists. The temperature ranged 15° on the grass, to 60°, in the middle of the day on the 7th and 8th, and then fell daily to 23° on the grass, on the 16th. On the following day the highest was 33°. It thus assumed a December temperature, averaging 40° to the 27th. After this period the cold became excessive; in the night of the 27th the minimum degree was 13°, and on the 31st it rose to 28°—a range of 17°. The same epidemic existed in the neighbouring village as in November, and those who had hitherto escaped became the victims of the disease. The disease chiefly attacked the improvident and badly habited. Catarrh and influenza were very prevalent in this month, evidently from the rapid changes of climate. In old people, influenza was not only severe, but fatal in some cases. Pleuritic and pneumonic diseases also came under notice amongst the working classes, requiring the most active treatment, and erysipelas occurred in three cases. Children also suffered from the changes, and from imprudent exposure to the severe cold. Bronchitic affections were common with them, and in the very young terminated fatally. In all the cases of fever, diarrhœa preceded the attack, producing the most severe exhaustion; the cases, unless early attended to, fell into low fever. One case of carbuncle occurred in this month in an old and very poor man; he ultimately did well. The casual diseases under treatment during this month were menorrhagia, nephritis, neuralgia, chlorosis, hypertrophy of the heart, syphilis, gonorrhœa, and some cutaneous affections.

"The same vacillating state of atmospheric pressure continued in the month of January as in the previous month, ranging from 30.30 to 28.80. The weather was, during twenty-three days, rainy and overcast, with fogs and hail. There were thirteen windy days; the amount of rain was 1.88 inch. The temperature of the month ranged between 50° and 18°. The transitions were not so sudden. This state of climate lessened the amount of illness amongst the poor. The cases of fever were reduced in number, but some deaths occurred. Rheumatism and pleurodynia were more prevalent with working men; and among children, bronchitic disease was unusually prevalent, from the careless habits of the parents in exposing them to the cold. Influenza was less frequent this month; three cases only came under treatment. Cynanche tonsillaris in females came under notice. Only one case of erysipelas occurred. No specific infectious disease occurred during this month. The casual diseases under treatment were epistaxis, abdominal tumour, hysteria, tic douloureux, hydrothorax from diseased heart, asthma, and some cutaneous eruptions.

"The month of February brought comparatively spring weather. The little fluctuation of the barometer during the month, being only 0.7, the temperature varying daily but a few degrees, the little wind, and the paucity of rain, produced great changes as to the healthiness of the month. Of the epidemic low fever, only one case occurred during the month, and it is progressing favourably towards recovery. One case of hooping-cough occurred from exposure to cold. Two cases of spasmodic croup were fatal in the same family, after a very few hours attack; a third case occurred in an elder daughter, in whom the most active treatment was pursued, and she recovered. Only one case of influenza occurred during the month, and catarrhs and coughs were less prevalent. Erysipelas now and then came under notice; in one case, in which it attacked the leg of an elderly gentleman, it arose from a slight accident, in a habit exceedingly predisposed to it. Ague was under treatment in two cases in this month; it occurred in labouring men who had been working in the river. Pleurodynia now and then came under our care, but was soon relieved. Now and then a case of bronchitis was presented. I consider this month to have been unusually healthy. The casual diseases were valvular disease of the heart, ascites, tic douloureux, syphilis, hydrocele, spermatorrhœa, fracture, cardialgia, gout, and impetigo.

"From information I receive from the veterinary surgeons, I find that much disease existed during this quarter amongst the horses—sore throats, glanders, and pneumonia. The diseases have been very serious, attended with fatality ; the same complaints have attacked the herd stock. Fevers have also been prevalent amongst them. But during the last month a considerable change has taken place ; they are become more healthy. No complaint exists amongst the sheep, and there is a good prospect for the yeaning time. There is no lack of food for them. The few lambs already yeaned are remarkably strong and healthy."

*Record of Deaths in Twenty-one Parishes (population* 9,574) *from the Registrar's Book.*

| *December.* | | *January.* | | *February.* | |
|---|---|---|---|---|---|
| Cancer of the face .. | 1 | Decay of nature .... | 1 | Pneumonia ........ | 1 |
| Croup ............ | 1 | Bronchitis ........ | 1 | Diarrhœa.......... | 1 |
| Consumption ...... | 2 | Atrophy .......... | 2 | Decay of nature.... | 6 |
| Accidental death.... | 1 | Consumption ...... | 3 | Dropsy............ | 1 |
| Pneumonia ........ | 1 | Fever .... ....... | 1 | Lumbar abscess .... | 1 |
| Fever ............ | 1 | Hæmorrhage ...... | 1 | Spasmodic croup .. | 2 |
| Decay of nature .... | 3 | Diseased heart .... | 1 | Valvular disease of heart .......... | 1 |
| Dropsy............ | 2 | Tabes mesenterica . | 1 | Fever ............ | 4 |
| Premature birth.... | 1 | Typhus .......... | 1 | Consumption ...... | 1 |
| Anæmia .......... | 1 | | | | |
| Debility .......... | 1 | | | | |
| Diseased liver...... | 1 | | | | |
| | 16 | | 12 | | 18 |

*Dudley.*—Mr. Houghton writes : "The predominant feature in the health of Dudley during the thirteen weeks ending February 27th has been a widely spread eruption of small pox, attacking all classes and all ages of persons, and proving fatal to fifty individuals. Of the fifty persons who died, eleven are said to have been vaccinated and twenty-six not. No report has been made on this point on the thirteen remaining cases. The proportion of deaths after vaccination, according to this statement, is enormous, being more than one in three of the whole deaths. There is good reason to doubt the efficient vaccination in these cases. In one case under my own observation modified small pox occurred a second time after vaccination ; the patient was about 60. In some cases the eruption was preceded for some days by a rash, almost exactly resembling measles in its appearance, but not accompanied by the usual catarrhal symptoms. Scarlet fever has proved fatal in four cases only ; one of these being quite irregular in its manifestations, and of intense malignity. Four days of extreme constitutional disturbance, the principal feature of which was agonising spasmodic pain in the loins, ushered in a rash, so full that it was with difficulty diagnosed from erysipelas when it first appeared, the throat affection being slight; low delirium followed with extreme prostration; on the third day of the rash she miscarried; on the fourth, petechiæ appeared early in the morning; and it is no figure of speech to say that she died *purple* in the evening. There was nothing in the place or the position of the patient to account for this malignity. Fever and diarrhœa have as usual prevailed during the whole quarter. During this period, 333 deaths were registered; the average of the spring quarter of the last eight years being 295; 85, or 1 in 3·915, arose from zymotic diseases. In the corresponding period 16,009 deaths were registered in the metropolis, 2,835 being from zymotic diseases, or 1 in 5·646. Thus the sanitary condition of Dudley contrasts very unfavourably even with overgrown London."

*Barrowden.*—This district for the quarter has been very healthy in comparison with last year. Hooping-cough still continues to spread. Diarrhœa, after the break up of the frost, was very prevalent for a fortnight. The wind has been west all winter, with a few days' exception.

*Wisbeach.*—Mr. Hole states that there has been more typhus in this town during the last quarter, coming under his own notice, than at any other period of his residence here.

*East Dereham.*—Three cases of small pox occurred, all after vaccination; these were very slight, but plainly marked. Two patients were adults and one a youth. (Dr. Vincent.)

*Pontesbury.*—Two cases of puerperal fever occurred at the same time in February. Scarlet fever, measles, and typhus have been very prevalent through the neighbourhood. (Mr. Eddowes.)

*Burton-on-Trent.*—Dr. Thomson says: "By far the most frequent diseases during the last quarter, so remarkable for its atmospheric vicissitudes, have been the various forms of catarrh and bronchitic affections, in addition to pneumonic and pleuritic cases. Neuralgia, especially sciatica, has been frequent; but the most unusual addition to our diseases has been jaundice, occurring chiefly among children under ten years of age. Case after case has occurred, and, in one instance, two cases in one family. Girls have been more generally attacked than boys, at least under my observation. The disease has always yielded readily to treatment."

*Oswestry.*—Mr. Cartwright records that "small pox was imported from Staffordshire, and confined itself to the immediate neighbourhood of the original case. Two of the subjects had not been vaccinated; the others had the disease modified."

*Gainsborough.*—Dr. Mackinder writes: "We have had rather more than the usual amount of sickness this quarter, but no epidemic. Since the end of January, I have not had a single case of typhoid or continued fever; but a few children have been affected with remittent fever. On each side and passing under the house in which the best marked case occurred, there is a common sewer, the stench from which is often intolerable. There have been a few cases of diarrhœa, dysentery, erysipelas, bronchitis, tonsillitis, and measles. Four cases of scarlet fever occurred in one house, where the poison seemed to originate and exhaust itself. I have had three cases of phlegmasia dolens; one was a poor woman far advanced in phthisis, and the other two were very anæmic subjects. I have had one case of carbuncle and one of croup. The importation and propagation of small pox, of which there have been three cases, have created a little alarm. The first case was a young sailor, who disembarked at Liverpool, from New Orleans, on January 14th. He remained at Liverpool two days, and then proceeded to Hull, where, during his brief sojourn of a couple of days, he saw a friend who was recovering from small pox. He then came to Gainsborough, and ten days after his arrival was seized with the disease. At the expiration of ten more days a brother became affected, and then the mother, aged 57, was attacked. All the cases were mild ones. The young men had been vaccinated; but the mother could not certify to the inoculation of herself. Another brother, who had sailed in the same ship from New Orleans, but had not been to Hull, escaped; nor has he yet suffered from his exposure to the infection at the house of his parent. I have had a case of tertian ague in a village four miles hence. My patient was an old woman who had been ill for some weeks. About a yard from her dwelling there was a filthy open drain; this, on being reported, was ordered by the board of guardians to be immediately cleaned out. The house is one of those low, dark, mud-walled, thatched, indefinable mediæval structures which are a disgrace to any civilised community."

The following meteorological report is furnished by Mr. Dyson: "The severe weather with which the month of December opened continued only for a few days; a thaw commenced on the 5th, and the remainder of the month was changeable to the end. The weather continued unsettled throughout the quarter; showers, fog, and sleet alternating. The last night in January was intensely cold; the thermometer fell to 12°, and to 19° the following night; in other respects the temperature was above the average nearly all the quarter. The amount of rain which fell in December was 1·46 in.; in January, 2·44 in.; and in February, ·85 in. Ozone was freely developed from the 5th to the 14th of December, the whole of February, but very slightly in the month of January. Dense fogs prevailed the last fortnight of February. Throughout the quarter the weather may be described as 'unsettled' till the 5th of February."

*Liverpool.*—Mr. Bickerton forwards the following report, compiled by the excellent medical officer of health to the borough, Dr. Duncan, and read by the clerk to the Health Committee: "During the year ending 27th December last, 11,574 deaths were registered in the borough, being 931 fewer than in the preceding year, and giving a lower ratio of mortality than has ever previously been recorded in Liverpool. In 1850, as a consequence of the high mortality from cholera in the previous year, the deaths were fewer in number; but allowing for increase of population, the mortality of 1856 was a fraction lower than that of 1850. During the previous ten years, the deaths averaged 36 annually in every 1,000 of the population; in the last five years of this term, they were 31 in 1,000; last year, they were 27 in 1,000 of the population. From diseases of the zymotic class, the deaths were 3,070: the average of the previous five years being 3,510. With the exception of scarlatina in the first and last quarters of the year, and diarrhœa, which caused a large infantile mortality in the hot weeks of summer, no disease of this class was more than usually prevalent. Typhus, which was formerly the opprobrium of Liverpool, and the prevalence of which is usually considered a test of a low sanitary condition, caused only 342 deaths, being 61 fewer than the number in the previous year, which was itself the lowest ever recorded up to that time. It is satisfactory to know that this diminished mortality from fever is not, apparently, dependent upon casual or accidental causes; for if an average of years be taken for comparison, the decrease is most obvious and decided. In the four years (1837-40) immediately subsequent to the passing of the Registration Act, one in about 450 of the inhabitants of Liverpool died from fever annually; while in the last four years (1853-56), the yearly deaths were not more than one in 1,035. In the metropolis, during the same periods, the fever deaths were one in 690, and one in 987, respectively. The contrast is gratifying, and may be taken as an index of the extent to which sanitary operations have been carried out in the respective places.

Last year (1856), the deaths from fever in Liverpool were one in 1,235; and in the metropolis, one in 989 of the population. Diarrhœa caused 766 deaths, of which 661 were in cases of infants or children; and nearly one-half of the whole took place in six weeks between the 2nd August and the 13th of September. Of 643 fatal cases of scarlatina, 428 occurred in the March and December quarters; hooping-cough caused 444 deaths; measles, 310; croup, 158; small-pox, 98—of which 24 were reported as after vaccination, the remainder being unvaccinated; syphilis, 65; dysentery, 60; infantile and remittent fever, 55; erysipelas, 46; thrush and canker, 29; cholera, 26; purpura, 9; scurvy, 5; mumps, 5; influenza, 4; chicken-pox, 4; ague, 1. From hooping-cough, remittent fever, cholera, and purpura, the deaths were exactly the same in number as in the previous year; in the other diseases of this class there was a decrease, with the exception of diarrhœa, already mentioned, and of small-pox, which caused four deaths more than in the year preceding. This disease at present shows some disposition to increase, the deaths in the last five quarters having been respectively 15, 17, 18, 28, and 35; and in the first eight weeks of the present year, 32. Disease of the lungs caused 3,420 deaths, including 1,406 from consumption. The average of the preceding eight years was 3,513, including from consumption, 1,470.

"The mortality in each quarter was as follows, viz.

| | All causes. | | Disease of lungs and consumption. |
|---|---|---|---|
| March quarter............ | 3,145 | .... | 1,102 |
| June quarter ............ | 2,626 | .... | 868 |
| September quarter........ | 3,041 | .... | 603 |
| December quarter ........ | 2,762 | .... | 847 |

The deaths from consumption in the four quarters consecutively were 408, 385, 311, 302.

"Inquests were held on 643 cases of sudden or violent deaths (449 male, 194 female—233 children, 410 adults). Of these, there were from excessive drinking, 43; starvation and cold, 1; want of assistance at birth, 1; accidentally poisoned, 3; burns and scalds, 86; hanged for murder, 1; suffocation, 74; drowning, 63; accidentally killed, 188; murder, 16; manslaughter, 18; natural disease (specified), 13; ditto (not specified), 115; suicide, 21—of which by hanging and strangling, 9; drowning, 1; poison, 5; cut throat, 6; jumping from window, 2. Of the suicides, 18 were males and 3 females (two of these unmarried). The poison used was in two cases laudanum, in one prussic acid. Ten of those who committed suicide were persons in good circumstances. The extreme ages were 19 and 70. Of the cases of accidental drowning, 34 occurred in the docks, 18 in the river, and 9 in the canal. Of those accidentally killed, 24 were by carts and carriages, and 7 by railway accidents. Sixty-five deaths (one less than in the previous year) were ascribed to the direct effects of intemperance; besides which, seven fatal accidents occurred to persons in a state of intoxication, five of these being married women. Of the entire number, 45 were males, and 27 females; and of these last, 18 were married, 3 were widows, 3 unmarried, and 3 unknown. Eight were from 23 to 30 years of age; 22 from 30 to 40; 28 from 40 to 50; 12 from 50 to 60; and 2 were respectively 65 and 68. A married woman, aged 37, died from the long continued use of opium.

"The rate of mortality, of course, varied much in the different districts of the borough, ranging from 21 in 1,000 in North Toxteth ward, to 39 in 1,000 in Vauxhall; the latter, as has invariably been the case, presenting the highest district death rate. The following table, in which corrections have been made for deaths in workhouses and hospitals, shows the relative mortality of the different districts :—

| Wards, etc. | Deaths. | In 1000 inhabitants |
|---|---|---|
| Vauxhall | 863 | 39·4 |
| St. Anne's | 738 | 35·5 |
| Exchange | 475 | 33·4 |
| Scotland | 2061 | 31·4 |
| Lime Street | 425 | 29·0 |
| St. Paul's | 379 | 28·7 |
| St. George | 482 | 28·4 |
| Castle Street | 199 | 27·0 |
| Pitt Street | 265 | 25·7 |
| Abercromby | 535 | 23·3 |
| St. Peter's | 184 | 21·7 |
| Rodney Street | 421 | 22·1 |
| Workhouse and Fever Hospital | 908 | — |
| Hospitals | 346 | — |
| Parish of Liverpool | 8,181 | 29·9 |
| Everton and Kirkdale | 1,065 | 24·7 |
| South Toxteth | 867 | 24·0 |
| West Derby | 588 | 21·1 |
| North Toxteth | 661 | 20·8 |
| West Derby Workhouse and Fever Hospital | 196 | — |
| Kirkdale Gaol | 3 | — |
| Industrial Schools | 9 | — |
| Out Townships | 3,393 | 22·7 |
| Borough of Liverpool | 11,574 | 27·3 |

"Of all who died during the year, 6,062 were males, 5,512 females; the death rate being for the former 296, and for the latter 253 to 1,000. The ages at death were: below 1 year, 3,017; 1 to 2, 1,519; 2 to 5, 1,473; 5 to 15 years, 746; 15 to 20, 252; 20 to 40, 1,668; 40 to 60 years, 1,640; 60 to 80,

1,048; and above 80 years, 178; unknown, 33. Of 16 persons who died at the age of 90 and upwards, 14 were females; one of these, formerly a domestic servant, being recorded as 'about 100 years' old at the time of her death.

"The births registered during the year in the parish were 9,270, being 80 more than in the preceding year, and giving an excess of 1,089 over the deaths. In the out-townships, the births were about 5,584, 240 more than in the preceding year, and giving an excess over the deaths of 2,191. The total births in the borough were thus 14,854, and the excess of births over deaths 3,280, being about double that amount to be added to the population of Liverpool by immigrants from other places. The births have been in the proportion of 35 to every 1,000 inhabitants; in the metropolis, the proportion was 33 in 1,000."

Mr. Bickerton makes also the following weekly statements: "Dec. 6th, 1856. The extreme cold that set in the previous week had a sensible effect in increasing the mortality. There were registered 244 deaths, being 35 more than in the previous week, and more than in either of the last 13 weeks. Diseases of the lungs caused 92 deaths; 22 being from phthisis, the remaining 70 from inflammatory diseases, being a greater number in any week since the intense frost in the spring of 1855. Scarlatina caused 18 deaths; measles, 4; small-pox, 5 (three not previously vaccinated); typhus, 8; croup, 10; hooping-cough, 8; syphilis, 2. There were more deaths from croup than ever before registered in Liverpool: 144 of the deaths occurred in children, 100 in adults. The mean temperature was low, 35·8, until Saturday; on Thursday it was 29·5; on Saturday it rose to 48·75, the average being about 53·5, consequently the temperature for the week was nearly 7 degrees below the average for the same week during last ten years. Rain fell every day except Sunday; total amount ·045 inch.

"Dec. 13th. There was a great decrease in the mortality of the town owing to the much milder temperature that prevailed. The number of deaths registered was 179, being 65 less than the corrected average for preceding eight years. Scarlatina caused 17 deaths; measles, 4; small-pox, 0; typhus, 8; hooping-cough, 4; croup, 4; erysipelas, 2; diarrhœa, 1. There were 90 deaths below 15 years of age, and 21 above 60. The mean temperature rose from 35·8 to 51·9.

"Dec. 20. 196 deaths were registered, being 42 less than the average for the corresponding week of previous eight years. Scarlatina caused 10 deaths; measles, 9; small pox, 4 (3 not previously vaccinated); typhus, 10; hooping-cough, 4; influenza, 1; syphilis, 1. Diseases of lungs 74 (32 from consumption).

"Dec. 27. The deaths registered during the past seven days were 191, being 55 less than the average. Scarlatina caused 16 deaths; measles, 5; small pox, 7 (3 not vaccinated); typhus fever, 10; hooping-cough, 8; scurvy, 1, being the only death from this disease during the last fourteen months. Diseases of lungs caused 64 deaths; 28 in children, and 36 adults. The weather was unsettled wet till the 24th, then thunder and lightning, followed by a heavy fall of snow and frost.

"During the three months ending 31st Dec., 1856, the deaths from all causes were 2,759; including 699 sudden, violent, and deaths in hospitals. The corrected average for the past nine years, for the same quarter, is 3,379. Liverpool is rapidly rising from being one of the most unhealthy to one of the most healthy towns in the kingdom. Zymotic diseases caused during the quarter 737 deaths, the average being 915. Of these, scarlatina caused 228; measles, 79; small pox, 35 (10 only having been vaccinated); typhus fever, 105; hooping-cough, 65; croup, 57; diarrhœa, 103. Diseases of the lungs, 835. In connection with these, it must be stated that the mean temperature of the quarter was higher than the average.

"Jan. 3, 1857. There was an increase in the mortality, 226 deaths having occurred, being 35 more than the previous week; the average for the week, however, was 250. Scarlatina caused 13 deaths; small pox, 1; hooping-cough, 9; diarrhœa, 7; ague, 1; measles, 11; typhus, 8; croup, 0; syphilis, 2. Diseases of lungs caused 81 deaths, owing to the very cold weather that prevailed from the 25th Dec., 1856, to Jan. 1, 1857.

"Jan. 10. 197 deaths were registered during the past week, being 73 less than the average. Scarlatina caused 9 deaths; measles, 12; small pox, 5 (2 only being vaccinated); typhus, 4; hooping-cough, 7; croup, 1; diarrhœa, 11; ague, 1. Diseases of lungs 61; delirium tremens, 1. The weather was fine and cold.

"Jan. 17. 218 deaths occurred, being 37 less than the average. Scarlatina caused 7 deaths; measles, 4; small pox, 8 (4 not vaccinated); typhus, 5; hooping-cough, 5; croup, 4; syphilis, 5; diseases of lungs, 79. The mortality from small pox was higher than in any week of the preceding five years.

"Jan. 24. There was another slight increase in the number of deaths. 241 deaths from all causes were registered; but this number does not reach the corrected average for the last nine years, nor can we be much astonished at this increase when we consider the very great and sudden changes in the temperature and general state of the weather during the past 30 days. There were 90 deaths from chest affections. Deaths from other causes, scarlatina, 17; hooping-cough, 7; typhus, 11; puerperal fever, 1; measles, 10; diarrhœa, 7; infantile remittent, 8; small pox, 3 (one not vaccinated); syphilis, 3.

"Jan. 31. There were 217 deaths registered during the week, being 33 less than the average. Scarlatina caused 6 deaths; measles, 8; hooping-cough, 6; typhus, 3; diarrhœa, 3; syphilis, 5. Diseases of lungs caused 89 deaths, 31 being from phthisis. 94 deaths occurred in children below five years of age, and 1123 in persons above five. The weather was unsettled and changeable, but wet and cold generally.

"Feb. 7. There were 238 deaths registered during the week, being 21 more than in the preceding week, but fewer by 14 than the corrected average. Scarlatina caused 10 deaths; hooping-cough, 2; measles, 6; small pox, 4; typhus, 2; diarrhœa, 2; syphilis, 2. From diseases of lungs the deaths were higher than in any week of the last 12 months, the number being 95. (37 from phthisis.) This was owing to the very severe weather, cold and changeable, which has been also very destructive to the old, 42 of the deaths being in persons above sixty.

"Feb. 14. There were registered during the week 231 deaths, being 10 below the corrected average. Scarlatina caused 9 deaths; measles, 10; small pox, 4 (three not vaccinated); typhus fever, 9; diarrhœa, 2; syphilis, 2. A woman aged 58 died from convulsions induced by spirit drinking. Diseases of lungs caused 102 deaths, a greater number than has occurred since the spring of 1855.

"During the latter weeks of February, and at present (March 2) there has been a species of cynanche tonsillaris existing in horses. I am told by one of our most respectable veterinary surgeons that some new cases have come under his care."

*York.*—There has been a greater amount of disease in February than in the preceding months, but this has not been very extensive. Hooping-cough, measles, and carbuncle have been very prevalent. There have also been several cases of cramp, which is not here of frequent occurrence. (Mr. Proctor.)

*West Auckland.*—Mr. Todd says:—"The principal epidemic diseases which I have had to treat during the last quarter have been measles, hooping-cough, and scarlet fever. Measles prevailed during the month of December, and in a large majority of the cases were complicated with a low form of inflammation of the bronchia and lungs. Many deaths resulted from this complication. The weather during the month of December was intensely cold. Hooping-cough prevailed during the same month, and, like measles, was in many cases complicated with a fatal form of pneumonia. During the months of January and February scarlet fever has, and still continues to prevail.

Many cases have been attended with very severe affection of the throat and the glands of the neck. Several cases have been followed by anasarca and effusion into the chest. Upon the whole the epidemic is of a very malignant nature, and many deaths have resulted. Many children suffer from a low form of pneumonia independent of measles and hooping-cough."

*Rothbury.*—Mr. Summers writes:—"The principally prevailing diseases in this district have been typhus among adults, and remittent fever among children. The cases of typhus have generally been marked at first by bilious symptoms; afterwards by great debility and a tendency to affection of the head. In this, and indeed in almost all diseases for some time past, great nervous depression appears to me peculiarly prominent; and I find that accordingly typhus patients bear stimulants almost from the commencement; and, even in inflammatory complaints, I have for some time noticed that patients bear very little depletion, and this notwithstanding that the general *physique* of the people of this district is considerably above the average as to robustness and general vigour. Of the children who have been attacked with remittent fever, some have been in houses where typhus was existing, others where there could have been no typhus *infection.* These remittent cases, however, have been marked, like those of typhus, by great depression and a tendency to affections of the head. In several cases I have noticed a remarkably distinct eruption of very fine and numerous spots all over the body; and in a case of a child between 2 and 3 years old, I could see nothing to distinguish it from a case of typhus in the adult, except that the remissions were, for the first week, tolerably distinct."

*Lerwick.*—Dr. Spence says:—"The winter commenced early; there were several heavy falls of snow, which, however, never lay long; the cold was not great, but the weather was extremely variable and boisterous, with frequent heavy gales; the prevailing winds were from the S. W. Acute articular rheumatism was unusually prevalent, as were also muscular pains and pleurodynia. Small pox was twice imported from Leith, the first time in January, and the second in February. The number of cases of this disease was not great, and there were no deaths. I did not see the case returned as typhus till near its termination, and am by no means certain that it was not a case of the typhoid stage of influenza. The case returned as puerperal fever was probably, more correctly speaking, tubercular peritonitis, and occurred in a woman in an advanced stage of phthisis. One woman died at the advanced age of 101, and the average age in the cases of death under my observation (exclusive of the case of longevity just mentioned) was 53."

